# Quantifying Temporal Dynamics of *E. coli* Concentration and Quantitative Microbial Risk Assessment of Pathogen in a Karst Basin

**DOI:** 10.3390/w17050745

**Published:** 2025-03-04

**Authors:** Shishir K. Sarker, Ryan T. Dapkus, Diana M. Byrne, Alan E. Fryar, Justin M. Hutchison

**Affiliations:** 1 Department of Earth and Environmental Sciences, University of Kentucky, Lexington, KY 40506, USA; 2 Department of Civil Engineering, University of Kentucky, Lexington, KY 40506, USA; 3 Department of Civil, Environmental and Architectural Engineering, University of Kansas, Lawrence, KS 66045, USA

**Keywords:** karst spring, Kentucky, QMRA, *E. coli* O157:H7, water quality, sensitivity analysis

## Abstract

Karst aquifers can be highly productive water sources but are vulnerable to contamination by pathogens because of integrated surface and subsurface drainage. Our study focuses on the karstic Royal Spring basin in Kentucky, encompassing urban and agricultural land uses. The city of Georgetown distributes treated water from Royal Spring to over 33,000 customers. We examined *E. coli* dynamics at Royal Spring from June 2021 through June 2022, assessing variability under wet versus dry weather conditions. We also used quantitative microbial risk assessment (QMRA) to estimate potential health risks from the pathogenic bacterium *E. coli* O157:H7. *E. coli* concentrations in weekly water samples varied from 12 to 1732.8 MPN/100 mL, with a geometric mean of 117.2 MPN/100 mL. The mean concentration in wet periods was approximately double that during dry conditions. Because the pathogen was not detected by quantitative PCR (qPCR), we conducted QMRA based on literature data for water treatment plant operations (occupational) and recreational activities near the spring. The median probability of annual infection was 5.11 × 10^−3^ for occupational exposure and 1.45 × 10^−2^ for recreational exposure. Uncertainty and sensitivity analyses revealed that health risks were most sensitive to the pathogen/*E. coli* ratio and ingestion rate. Although the pathogen was not detected by qPCR, the presence of *E. coli* suggests potential fecal contamination. This highlights the importance of continued monitoring and investigation of different detection methods to better understand potential health risks in karst systems.

## 1. Introduction

Waterborne pathogens, including bacteria, viruses, and protozoa, can cause gastrointestinal (GI) illnesses such as diarrheal diseases and remain a global public health concern. Diarrheal diseases accounted for 1.3 million deaths as of 2015, with children under the age of 5 representing almost 0.5 million of these deaths [1], and an estimated 1.5 million deaths as of 2019 [2]. While high-income countries tend to have a lower burden of waterborne diseases overall, outbreaks stemming from microbial pathogens and deteriorating water systems in such countries have led to more research and public health initiatives in recent years to address this issue [3].

*Escherichia coli* (*E. coli*) serves as a crucial bacterial indicator of water quality, with its presence signifying potential fecal contamination [4]. The widespread health burden associated with diarrheal diseases has led to increased regulatory focus on *E. coli* monitoring in water systems globally [5]. The presence of *E. coli* in drinking water correlates with increased risk of both exposure to enteric pathogens and diarrheal diseases generally [6–8]. Although groundwater is commonly assumed to be less contaminated than surface water, a review of 649 outbreaks between 1948 and 2013 indicated that ~35.2 to 59.4 million cases of acute GI illness per year globally could be attributed to groundwater consumption [9]. As of 2000, up to 50% of drinking-water wells in the USA had some level of fecal contamination, and about 750,000 to 5.9 million illnesses per year were estimated to originate from contaminated groundwater [10].

Groundwater contamination by pathogens is especially concerning in karst aquifers due to their integrated surface and subsurface hydrology and short groundwater residence times [11]. While karst aquifers can be highly productive water sources, they are vulnerable to contamination due to rapid flow paths and limited natural filtration [12]. Microbial contaminants, including fecal bacteria, can easily enter karst aquifers through point recharge locations and be transmitted downstream [13]. Contaminant transport occurs at velocities up to hundreds of meters per hour, with less retardation or decay than in other aquifer types [14]. Bacteria can spread through karst systems while suspended in water or attached to sediments [15,16]. Sediment-bound bacteria in karst conduits can survive for weeks to months [17,18], acting as a reservoir for pathogens that can contaminate water supplies when sediments are stirred up by storms [19,20]. As a result, karst regions experience a high incidence of waterborne disease outbreaks when contaminated groundwater is used for drinking water supplies, whether through public systems or private wells [21]. Therefore, understanding temporal variations in pathogen concentrations is particularly crucial in karst systems, where rapid changes in flow conditions can significantly impact contaminant transport.

Karst landscapes commonly develop in carbonate rocks like limestone and dolomite and are characterized by dissolution features, including sinkholes, sinking streams, caves, and underground conduits [22]. Carbonate rocks prone to karst development underlie ~15% of the global ice-free continental surface, 40% of the land east of the Mississippi River in the USA, and up to 55% of the state of Kentucky [23–25]. In Kentucky, six public water systems rely on karst aquifers, collectively serving 157,573 persons directly and 173,792 indirectly as of 2020, representing 7.42% of the state’s 2019 estimated population [26,27].

The city of Georgetown in central Kentucky relies on a karst aquifer as its main water source. Georgetown Municipal Water and Sewer Service, which served 33,075 people as of 2020, draws water from Royal Spring [26]. Royal Spring is recharged by Cane Run, an ephemeral stream with mixed land use in its watershed, including agriculture and urban areas [28]. Prior studies have shown that the Royal Spring basin is significantly impaired by *E. coli* and other fecal indicator bacteria. Contamination originates primarily from anthropogenic sources such as sanitary sewer leaks, failing septic systems, agricultural and urban runoff, and leaching from waste material [28–30], although wildlife (e.g., waterfowl, deer, raccoons, and beavers) can also be substantial sources of fecal bacteria [28]. Sewage leaks have also been detected in the nearby city of Lexington’s Wolf Run watershed [31], part of the recharge zone for the city’s McConnell Springs [32]. Some rural Kentucky homeowners have used contaminated springs for water [33,34], which have been associated with enteric disease transmission in some cases [35]. However, the human health risks from exposure to pathogens have not been quantified for any of the public water systems in Kentucky relying on karst aquifers.

Exposure to waterborne pathogens can occur through drinking contaminated water or contact during water activities. Health risk from waterborne pathogens (for our case pathogenic *E. coli*) is usually quantified by the annual probability of infection or illness and diseases burden (DB) [36]. The probability of infection/illness and DB of *E. coli* exposure can be estimated using quantitative microbial risk assessment (QMRA) [37–40]. QMRA is a modeling framework used to estimate risk (e.g., as probability of infection, probability of illness) based on measured pathogen concentrations and dose–response models [36]. The framework consists of four steps: hazard identification, exposure assessment, dose–response relationship, and risk characterization [36]. QMRA can account for multiple pathogens and exposure pathways (including ingestion, inhalation, and skin contact) to quantify and compare the health risks associated with each route [41]. Because of its flexibility and quantitative nature, QMRA can inform public health decisions and policies by estimating location-specific illness risks related to pathogens and support water safety management [36,42].

While QMRA has seen wide adoption for drinking water and recreational water quality assessment in many contexts globally, very few studies have been conducted in karst settings. Stupar et al. [40] assessed microbial water quality of six karst springs in Romania and estimated the risk of GI illness for both adults and children using QMRA. Those authors inferred a high daily and annual risk of infection, with minimum and maximum values of 0.24 and 1.00, respectively, for five of the six springs studied [40].

This study builds upon recent work by Dapkus and colleagues [43,44] that established fundamental relationships between *E. coli* concentrations and hydrometeorological conditions in the Royal Spring system. While that study focused on precipitation-discharge dynamics and their influence on *E. coli* transport, as well as evaluating tryptophan-like fluorescence as a potential real-time proxy for *E. coli*, our current work extends this understanding to quantify potential public health risks through QMRA. In this study, we aim to quantify (i) *E. coli* dynamics in Royal Spring over a 1-year monitoring period, assessing variability under wet vs. dry weather conditions, (ii) estimate potential health risks from recreational and occupational exposure to *E. coli* using QMRA, and (iii) identify the groups at highest risk from exposure to water at Royal Spring. Uncertainty and sensitivity analyses are incorporated using Monte Carlo simulation and Spearman’s rank correlation, respectively, to understand the modeled health risks.

## 2. Materials and Methods

### 2.1. Study Area

**Figure 1.**
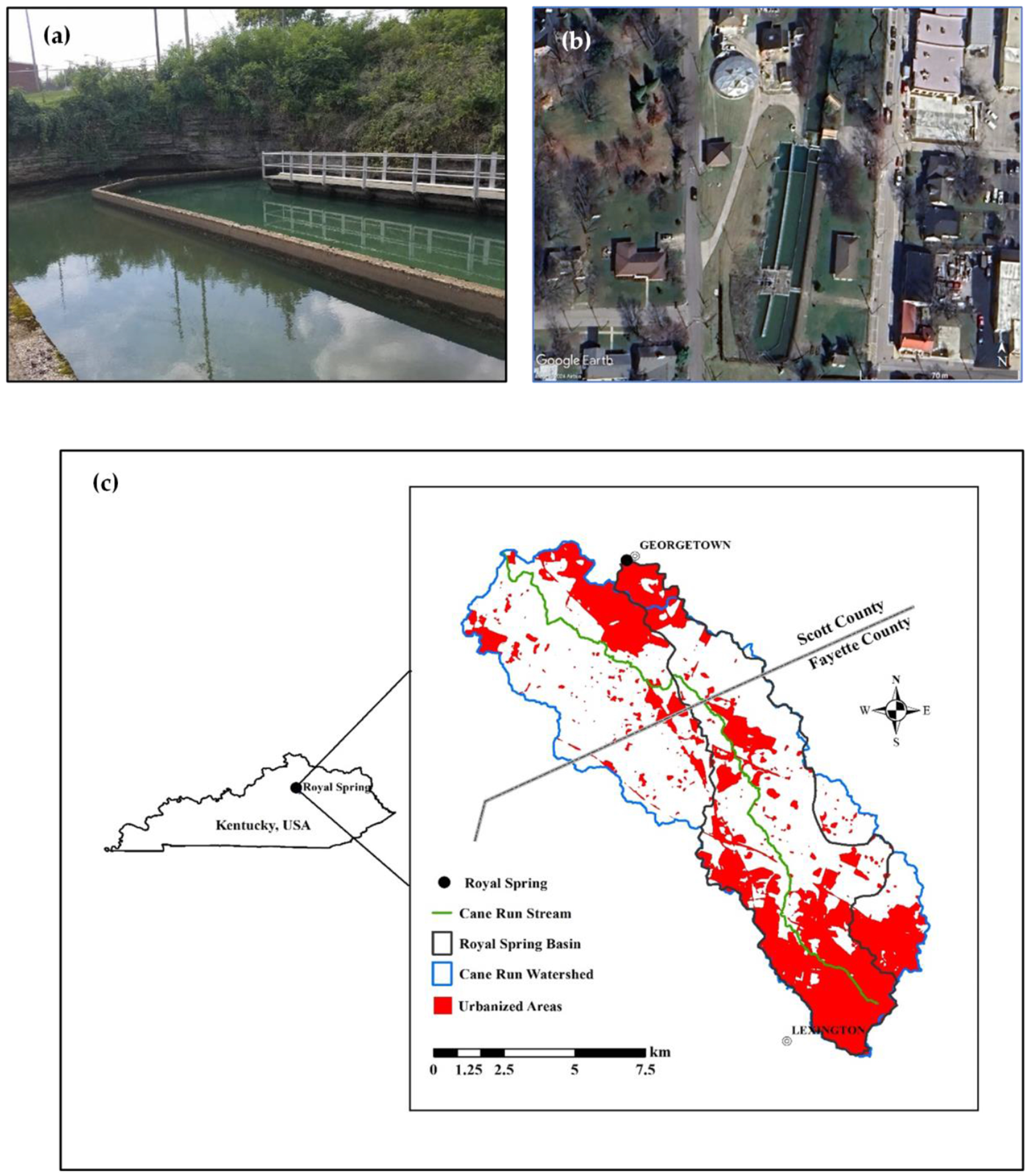
(**a**) View of Royal Spring showing the orifice and water treatment intake basin; (**b**) aerial view of Royal Spring and water treatment plant (modified from Google Earth); (**c**) map showing the Royal Spring basin within Cane Run watershed, and urbanized areas in Scott and Fayette counties, Kentucky, USA (modified from [44] with permission).

The Royal Spring groundwater basin is situated in the Inner Bluegrass physiographic region of central Kentucky, encompassing parts of Fayette and Scott counties (Figure 1). The basin drains an area of ~58 km^2^ and is characterized by a well-developed karst landscape formed on Ordovician limestones [45]. The geology is dominated by the Lexington Limestone, which has extensive karst development, including numerous sinkholes, sinking streams, and subsurface conduits [46]. A major karst conduit system connects surface inputs in the Cane Run watershed to Royal Spring [45,47].

The climate is humid-temperate, and precipitation is relatively evenly distributed throughout the year [48,49]. For the period 2001–2020, at Blue Grass Airport in Lexington (38.0408° N, 84.6058° W), the average annual air temperature was 12.5–14.8 °C, and the average annual precipitation was 851–1828 mm/year [50].

Land use in the Royal Spring basin is mixed, with ~40% of the watershed located within the urban area of Lexington, while the lower ~60% consists predominantly of horse farms and other agricultural land [45]. Soils in the basin are generally thin, consisting mainly of silt loams like the Bluegrass–Maury complex on uplands and the Huntington and Dunning series in valley bottoms [51]. The combination of mixed land use, karst geology, and thin soils in the Royal Spring basin creates a complex hydrological system that is highly vulnerable to pollution. This setting necessitates the detailed study of *E. coli* dynamics and associated health risks as outlined in the current research.

### 2.2. Sample Collection and Analysis

Water samples were collected weekly from Royal Spring from 9 June 2021 to 22 June 2022, with three exceptions (13 October 2021, 29 December 2021, and 6 April 2022). Water samples were collected from a bridge across the stream channel ~30 m downstream from the spring orifice (Figure 1a,b). *E. coli* enumeration was performed using the IDEXX Colilert-18 method (IDEXX Laboratories, Westbrook, ME, USA). After 18 August 2021, samples were diluted when necessary to avoid exceeding the maximum detection limit of 2419.6 MPN/100 mL. Further details of *E. coli* sampling and analysis protocols are given by [44].

For pathogen analysis, samples were collected weekly beginning 14 July 2021, except for the three dates noted above when sampling did not occur. We filtered 500 mL of each sample (total 1 L water) through a 0.45-μm, 47-mm diameter membrane filter (Advantec, Dublin, CA, USA). Filters were stored in a −80 °C freezer (except for the first two samples, which were temporarily stored in a −20 °C freezer for <1 week) until DNA extraction was performed. DNA was extracted from filtered samples using a DNeasy PowerWater kit (QIAGEN, Germantown, MD, USA). In brief, filters were placed in manufacturer-supplied 5-mL tubes with beads and PW1 solution. The samples were vortexed (ThermoFisher Scientific, Waltham, MA, USA) on a horizontal adapter (QIAGEN) at maximum setting for 5 min. The supernatant (approximately 600 μL) was transferred to a clean 2-mL tube, centrifuged for 1 min at 13,000× *g*, and transferred again to a clean 2-mL tube. DNA was extracted using a QIAcube Connect following the manufacturer ’s standard protocol [52]. Quantitative polymerase chain reaction (qPCR) assays were performed in 20-μL reaction volumes using SsoAdvanced Universal Probes Supermix (Bio-Rad, Hercules, CA, USA) on a CFX Connect Real-Time System (Bio-Rad). The reaction volume included 10 μL Supermix, 5 μL sample, 0.5 μL each of the forward and reverse primers (final concentration 250 nM), 0.3 μL probe (final concentration 150 nM), and 3.7 μL DNase-free water. Assay-specific primers, probes, and annealing temperatures were based on previously published reports [53–55] (Table S1). The thermal cycler protocol included an initial 3-min denaturing step at 95 °C followed by 45 cycles of a 15-s, 95 °C denaturation step, and a 45-s, 60 °C annealing/extension step. Blank samples included filtered laboratory water samples collected at the time of the field samples. Results were quantified using gBlock standards (Integrated DNA Technologies, Iowa City, IA, USA; Table S1) and reported as gene copy (gc) per volume of water originally filtered.

The qPCR analysis targeted the *stx1* gene of pathogenic *E. coli* O157:H7. Results were below the detection limit (<5.8 gc/100 mL) across all samples. Consequently, we relied on enumeration of *E. coli* indicator bacteria using the IDEXX Colilert-18 method for subsequent analysis.

### 2.3. Discharge and Precipitation Data

Discharge data for Royal Spring were obtained from the U.S. Geological Survey (USGS) gauge (Station 03288110) located ~90 m downstream from the spring orifice [56]. Daily water withdrawal data were acquired from the Georgetown water treatment plant for 1 June 2021 through 1 June 2022 (data for the remainder of June 2022 were unavailable) [57]. The final discharge values used in the analysis were calculated by adding gauged discharge and water treatment plant withdrawal rates to account for the entire spring output. This approach was necessary because the USGS gauge measures flow downstream of the water treatment plant intake.

Precipitation data for the period from June 2021 through June 2022 were obtained from the Midwest Regional Climate Center for the Lexington Blue Grass Airport weather station [50]. This station, located ~19 km from Royal Spring, was selected due to its long-term record. Precipitation patterns are relatively consistent across this distance, as validated by prior studies in the region [32].

### 2.4. Data Analysis

*E. coli* measurements that fell outside the detection limits of the IDEXX Colilert test method (<1 MPN/100 mL or >2419.6 MPN/100 mL, including after dilution when applicable) were excluded from the analysis to avoid potential bias introduced by censored data at the extremes of the measurement range [58]. By focusing on measurements within the quantifiable range, we maintained consistent analytical precision across all data points and improved the reliability of our statistical analyses.

To evaluate water quality conditions with respect to Kentucky’s water quality standards for the primary contact recreation season (1 May through 31 October), we analyzed *E. coli* concentrations using the criterion specified by the Kentucky Administrative Regulations [59]. While the regulation includes two criteria—a geometric mean criterion of 130 MPN/100 mL, based on at least five samples, within a 30-day period and a statistical threshold value of 240 MPN/100 mL not to be exceeded in 20% or more of the samples—our sampling frequency did not meet the requirements for geometric mean calculations. Therefore, we focused our analysis on comparing individual *E. coli* measurements to the 240 MPN/100 mL threshold. We calculated monthly exceedance frequencies by determining the percentage of samples exceeding this threshold during the recreation season. For the entire monitoring period, we calculated the geometric mean to characterize the central tendency of bacterial concentrations, accounting for the log-normal distribution typically observed in bacterial data [60].

None of the daily rainfall data were missing, but approximately 10% of the observations were recorded as trace amounts (designated as “T” in the original data). To quantify these trace precipitation events for statistical analysis, we assigned a value of 0.0025 inches (0.0635 mm) to all trace observations, representing half of the minimum measurable threshold (0.005 inches [0.127 mm]) as defined by the National Weather Service [61]. Antecedent moisture conditions (AMC) play a crucial role in karst hydrology, influencing the response of karst springs to precipitation events [13]. AMC can affect the storage and transmission of water through the epikarst and vadose zones, ultimately impacting spring discharge and water quality [62]. To account for this, we calculated the 48-h AMC by summing the rainfall from the 2 days preceding each sampling event. Sampling events with AMC ≥ 0.5 inches (12.7 mm) of rainfall were classified as wet, while those with AMC < 0.5 inches were classified as dry. This threshold aligns with the Kentucky Pollutant Discharge Elimination System regulations for stormwater management [63], which consider rainfall events ≥ 0.5 inches as significant for water quality monitoring purposes.

### 2.5. Statistical Analysis

We employed multiple statistical techniques to analyze the relationships between *E. coli* concentrations and environmental variables in the Royal Spring basin. We calculated summary statistics for all variables, including measures of central tendency and dispersion.

To predict the temporal variation of *E. coli* concentrations, a multiple linear regression (MLR) model was created with *E. coli* as the response variable and discharge, 48-h antecedent rainfall, and spring temperature as explanatory variables. The model’s performance was evaluated using several criteria, including coefficient of determination (R^2^), overall model significance (F-statistic and associated *p*-value), and individual predictor significance (t-statistics and *p*-values).

To compare *E. coli* concentrations between dry and wet conditions, we calculated geometric means for each AMC category. The Wilcoxon rank-sum (Mann–Whitney) test was implemented to assess whether there were significant differences in *E. coli* concentrations between these two conditions. This non-parametric test was chosen due to its robustness in handling non-normally distributed data, which are common in environmental studies [58]. A box plot was created to visualize the distribution of *E. coli* concentrations across AMC categories. All statistical analyses were conducted using R software (version 4.1.2) [64], with a significance level of *α* = 0.05.

### 2.6. Quantitative Microbial Risk Assessment

**Figure 2.**
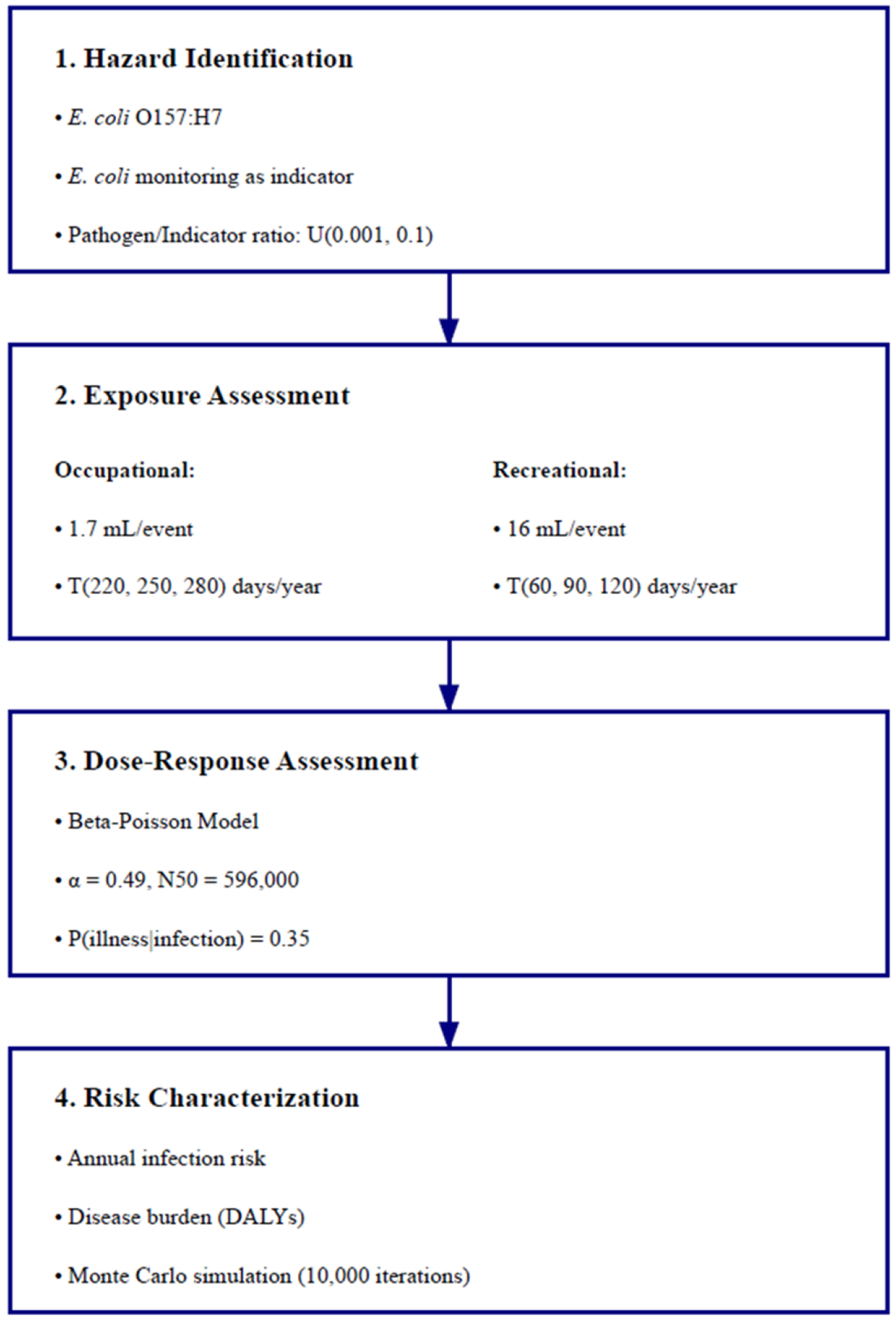
QMRA framework for evaluating health risks from *E. coli* O157:H7 in the Royal Spring basin. U and T represent uniform and triangular distributions, respectively, used in the Monte Carlo simulations.

In this study, we implemented QMRA following the standard framework with four steps: hazard identification, exposure assessment, dose–response relationship, and risk characterization (Figure 2). The analysis was performed using R software with Monte Carlo simulations to propagate uncertainty in pathogen-to-indicator ratios, exposure volumes, and dose–response parameters, which are explained in the following sections.

#### 2.6.1. Hazard Identification

Pathogens are often quantified using molecular methods, which are much more expensive and time-intensive than methods for quantifying fecal indicators such as *E. coli*. While our qPCR analysis did not detect the targeted pathogen, the consistent presence of *E. coli*, identified through the IDEXX Colilert test, indicated potential fecal contamination in Royal Spring. Therefore, we implemented QMRA using pathogen-to-indicator ratios and focused on *E. coli* O157:H7 as a reference pathogen following accidental water ingestion during occupational and recreational activities leading to GI illness. Infection through *E. coli* O157:H7 can lead to symptoms ranging from mild GI upset to bloody diarrhea and, in some cases, hemolytic uremic syndrome [38].

#### 2.6.2. Exposure Assessment

The exposure assessment incorporated two primary scenarios based on local water activities in the Royal Spring basin: occupational exposure during water treatment plant operations and recreational exposure near the spring and downstream waterways.

Exposure volumes for both scenarios were based on accidental ingestion during typical activities. For occupational exposure, a volume of 1.7 mL/event (SD = 0.91) was used following World Health Organization (WHO) guidelines [42] and previous exposure studies [36]. This volume accounts for inadvertent ingestion through splashing during sampling at the spring orifice, aerosol inhalation, and hand-to-mouth transfer during routine operations. This scenario focuses on raw water exposure before treatment processes. For recreational exposure, 16 mL/event (SD = 13) was selected based on comprehensive swimming exposure studies and recreational water quality criteria [60,65]. This volume represents accidental ingestion during activities such as wading and water contact. Both volumes were modeled using lognormal distributions to account for variability in exposure patterns and uncertainty in measurements. The pathogen dose was calculated using the following equation:

(1) D=C×R×V

where D indicates the pathogen dose per exposure event, C is the measured *E. coli* concentration (geometric mean) during the monitoring period, R indicates the pathogen-to-indicator ratio, and V is the volume of water ingested per exposure event.

While various surface water and drinking water studies have assumed that 8% of detected *E. coli* are pathogenic [36,37], karst aquifer systems present unique challenges that warrant a different approach. The distinctive characteristics of karst systems, including preferential flow paths, rapid transport, and variable retention times, can affect bacterial transport and survival patterns [66], as evidenced by differential transport of different *E. coli* strains within the Royal Spring aquifer [67]. For our analysis, we assumed a uniform distribution (U(0.001, 0.1)) for the pathogen-to-indicator *E. coli* ratio, where the lower bound represents the possibility of lower ratios during baseflow conditions and the upper bound accounts for rapid recharge events. This more flexible probabilistic approach allowed us to capture uncertainty in pathogen concentrations at Royal Spring, which proved to be critical as demonstrated by our sensitivity analysis (Section 3.4), where the pathogen-to-indicator ratio was the most influential parameter driving risk estimates.

#### 2.6.3. Dose–Response Assessment

In our study, the dose–response relationship for *E. coli* O157:H7 was modeled using the approximate Beta-Poisson model [36]:

(2) $$\text{P}(\text{inf})=1-{\left[1+(\text{D}/\text{N}50)\left(2^{\left(1/\alpha \right)}-1\right)\right]}^{\left(-\alpha \right)}$$

where P(inf) is the probability of infection per exposure event, D is the pathogen dose per exposure event, *α* is the shape parameter (0.49), and N50 is the median infectious dose (596,000) [36]. These parameters have been validated through analysis of human outbreak data [68,69].

The probability of illness given infection (P(ill|inf)) was set to 0.35 for *E. coli* O157:H7 based on the literature review of Machdar et al. [70], allowing for the calculation of the probability of illness per exposure event as follows:

(3) P(ill)=P(inf)×P(ill∣inf)

While the model parameters were derived from clinical studies rather than environmental exposures, sensitivity analysis (described in Section 2.6.5) was used to evaluate the impact of parameter uncertainty on risk estimates.

#### 2.6.4. Risk Characterization

An integrated exposure assessment for risk characterization was performed using a dose–response relationship to estimate annual infection risks and disease burden. Annual probability of infection was calculated as

(4) $$\text{P}(\text{annual})=1-{\left(1-\text{P}(\text{inf})\right)}^{\text{n}}$$

where P(inf) is the daily probability of infection (e.g., infection per exposure event) derived from the Beta-Poisson model and n is the number of exposure events per year. We assumed one exposure event per day and modeled annual exposure frequency using triangular distributions to account for uncertainty in duration. For occupational exposure, we assumed a triangular distribution with a minimum of 220 days per year, maximum of 280 days per year, and a mode of 250 days per year, representing approximate variations around a standard work year. For recreational exposure, the frequency ranged from 60 to 120 days per year with a mode of 90 days, reflecting typical seasonal use patterns with uncertainty.

Disease burden was quantified using disability-adjusted life years (DALYs), calculated as

(5) DALY=P(annual)×P(ill∣inf)×DB

where P(ill|inf) is assigned the same value as in Equation (3) (0.35) and DB is the disease burden per case of illness (0.0013 DALYs/case for *E. coli* O157:H7) [36,42]. The calculated risks were compared to the WHO reference level of 10^−4^ infections per person per year (pppy) and the tolerable disease burden of 10^−6^ DALYs pppy [42] to evaluate the public health significance of the exposure scenarios.

Risk characterization was performed separately for occupational exposure (water treatment workers) and recreational exposure scenarios. For each scenario, both the annual probability of infection and associated disease burden were calculated. Results are reported as median values with 95% confidence intervals derived from the Monte Carlo simulations (see Section 2.6.5) to reflect uncertainty in the risk estimates. The relative public health significance of each exposure pathway was evaluated by comparing calculated risks to WHO health-based targets and between scenarios. This approach allows for the identification of high-risk exposure patterns and supports evidence-based recommendations for risk management.

#### 2.6.5. Uncertainty and Sensitivity Analysis

To account for variability and uncertainty in model inputs, Monte Carlo simulations were performed using R software with 10,000 iterations. Key parameters were modeled as probability distributions based on literature values and empirical data, as noted in Section 2.6.2. The pathogen-to-indicator ratio and exposure volumes were modeled using the distributions described above. Dose–response parameters were characterized using beta distributions for *α* and N50 derived from [36].

Sensitivity analysis employed Spearman’s rank correlation coefficients to identify which input parameters most strongly influenced uncertainty in risk estimates. This non-parametric approach was chosen to account for potential non-linear relationships between inputs and outputs. Parameters were ranked by their absolute correlation coefficients to identify the most influential factors. The analysis was performed separately for each exposure scenario to identify potential differences in key parameters between occupational and recreational exposures. This comprehensive uncertainty and sensitivity analysis framework provided insights into both the magnitude of uncertainty in final risk estimates and the relative importance of different model inputs in driving that uncertainty.

## 3. Results

### 3.1. Temporal Dynamics of E. coli and Environmental Parameters

**Figure 3.**
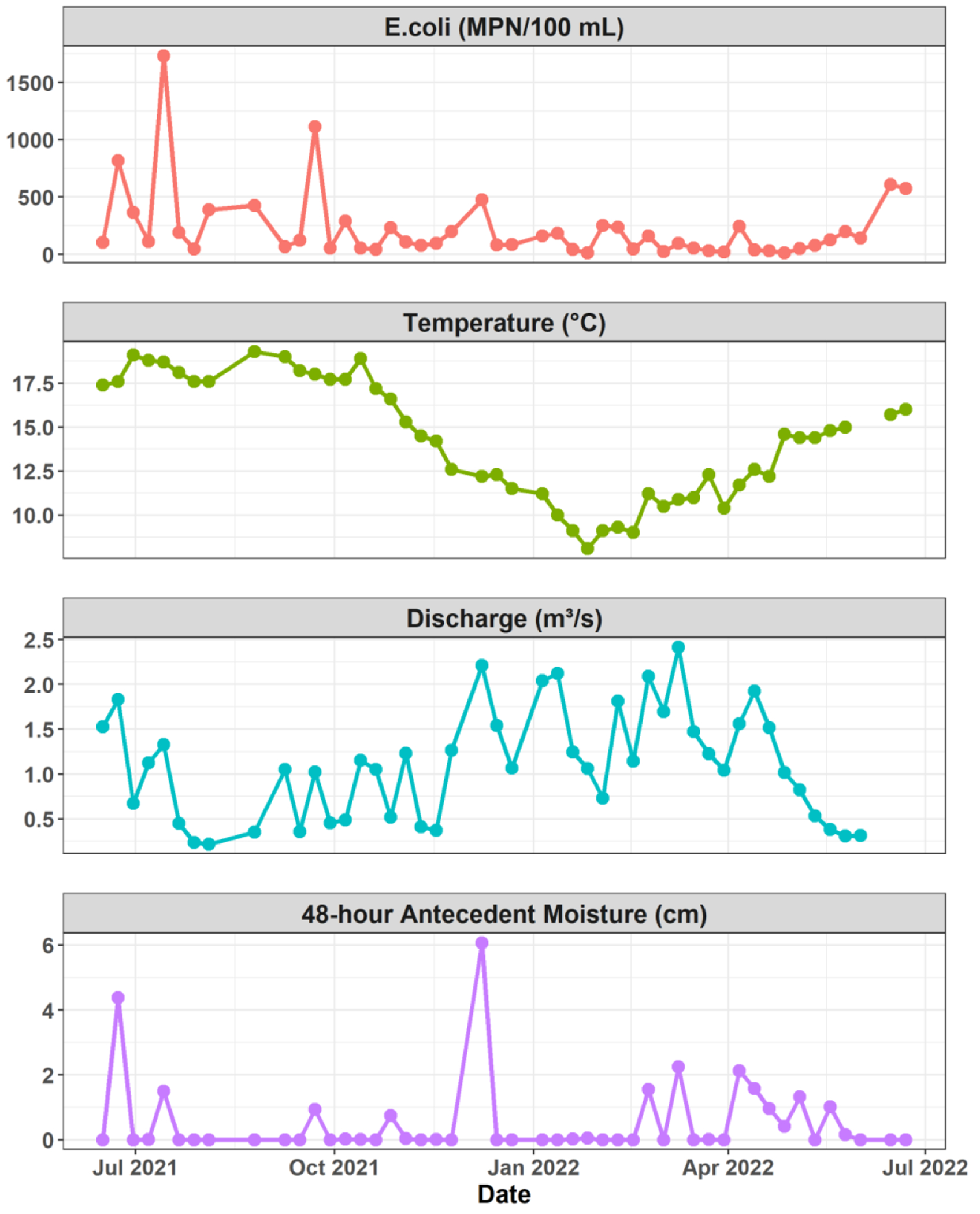
Time series of *E. coli* concentrations, water temperature, discharge, and 48-h antecedent moisture conditions during sampling events at Royal Spring from June 2021 to June 2022.

Water quality monitoring at Royal Spring from June 2021 to June 2022 revealed distinct seasonal patterns and considerable variability in both bacterial concentrations and environmental conditions (Figure 3). In addition to the 3 weeks when sampling was not conducted (see Section 2.2), on 11 and 18 August 2021, samples were undiluted, and *E. coli* concentrations exceeded the maximum detection limit (2419.6 MPN/100 mL). On 1 December 2021, the diluted sample fell below the minimum detection limit (1 MPN/100 mL). These three samples were thus excluded from the analysis.

For the remaining 48 samples, *E. coli* concentrations ranged from 12 to 1732.8 MPN/100 mL, with a median of 108.8 MPN/100 mL and a geometric mean of 117.2 MPN/100 mL. Spring discharge for monitoring dates varied between 0.2 and 2.4 m^3^/s (median 1.1 m^3^/s), while water temperature displayed expected seasonal fluctuations, ranging from 8.1 °C in winter to 19.5 °C in summer. The 48-h AMC ranged from 0 to 60.7 mm with a mean of 5.24 mm.

Analysis of monthly *E. coli* concentrations during the primary contact recreation (PCR) season (May–October) revealed varying frequencies of samples exceeding the 240 MPN/100 mL threshold. August exhibited the highest exceedance rate, with both samples exceeding the threshold, followed by June at 66.7% (four out of six samples). July, September, and October had 25% exceedance rates (one out of four samples each), while May had no exceedances out of four samples. Overall, during the recreation season, nine out of 24 samples (37.5%) exceeded the Kentucky PCR *E. coli* criterion, suggesting frequent water quality impairment in Royal Spring during warmer months when recreational use is most likely.

### 3.2. Influence of Wet Weather Conditions

**Figure 4.**
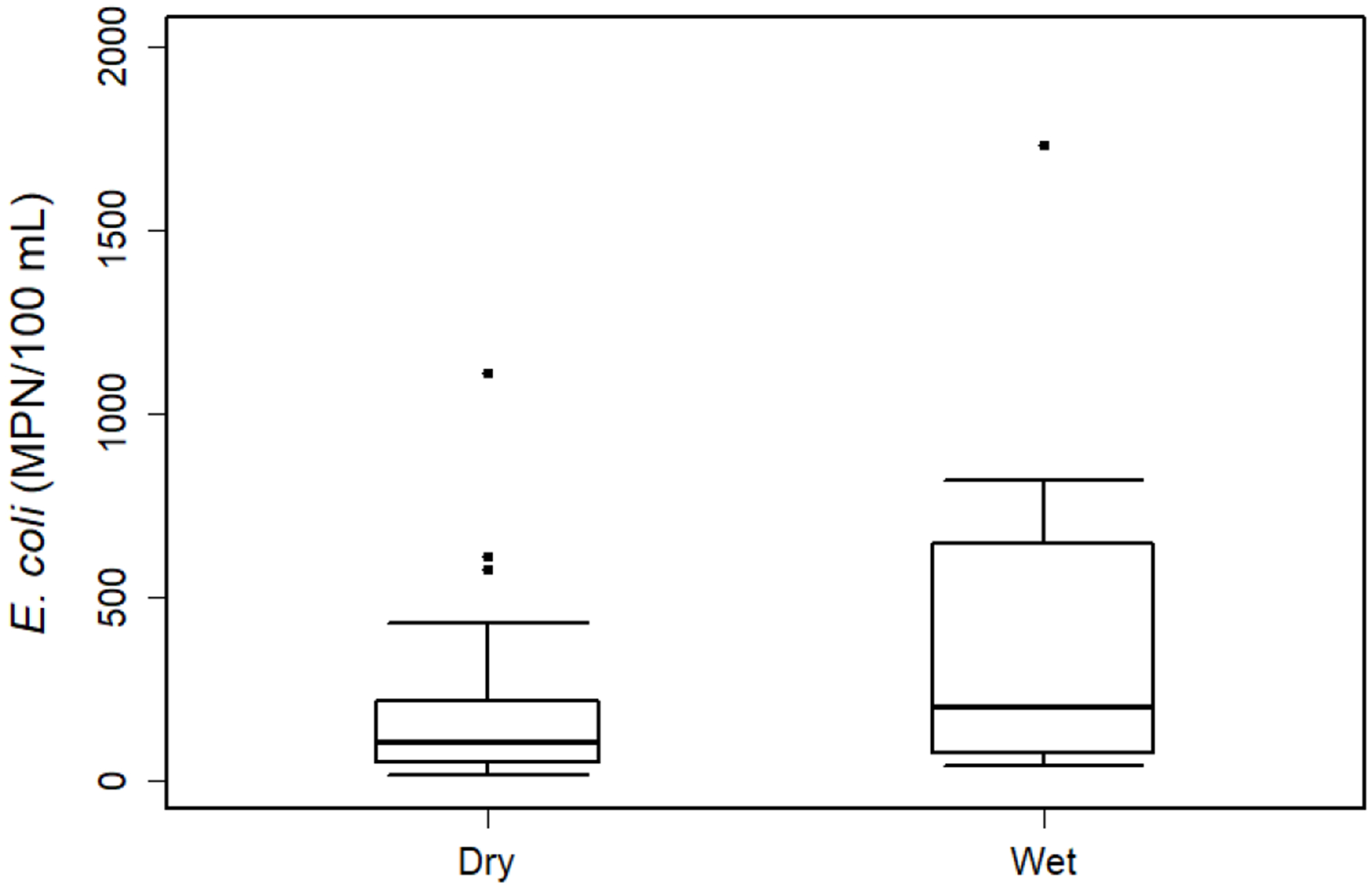
Distribution of *E. coli* concentrations at Royal Spring under dry and wet-weather conditions, as defined by 48-h antecedent moisture conditions. The plot displays the median (central line), interquartile range (box), and distribution range (whiskers). Individual points represent outliers exceeding 1.5 times the interquartile range.

Analysis of *E. coli* concentrations revealed notable differences between wet and dry weather periods. Samples categorized as wet conditions (AMC ≥ 12.7 mm) showed substantially higher *E. coli* concentrations, with a geometric mean of 215.8 MPN/100 mL compared to 103.7 MPN/100 mL during dry conditions (Figure 4). Despite this difference, the Wilcoxon rank-sum test did not indicate statistical significance at the 95% confidence level (*p* = 0.179), likely due to the limited number of wet-weather samples (8 of 48) in the dataset.

### 3.3. Environmental Controls on E. coli Variability

Multiple linear regression results revealed significant relationships between *E. coli* concentrations and environmental variables in the Royal Spring basin. The MLR model, incorporating discharge, 48-h AMC, and water temperature, explained 23.6% of the variance in *E. coli* concentrations (adjusted R^2^ = 0.2356, *p* = 0.002; Table 1). Two environmental variables emerged as significant predictors: spring temperature (*p* = 0.012) and 48-h AMC (*p* = 0.028), both showing positive relationships with *E. coli* concentrations. Discharge also exhibited a positive association with bacterial levels, but this relationship was not statistically significant (*p* = 0.581).

### 3.4. QMRA Results

**Figure 5.**
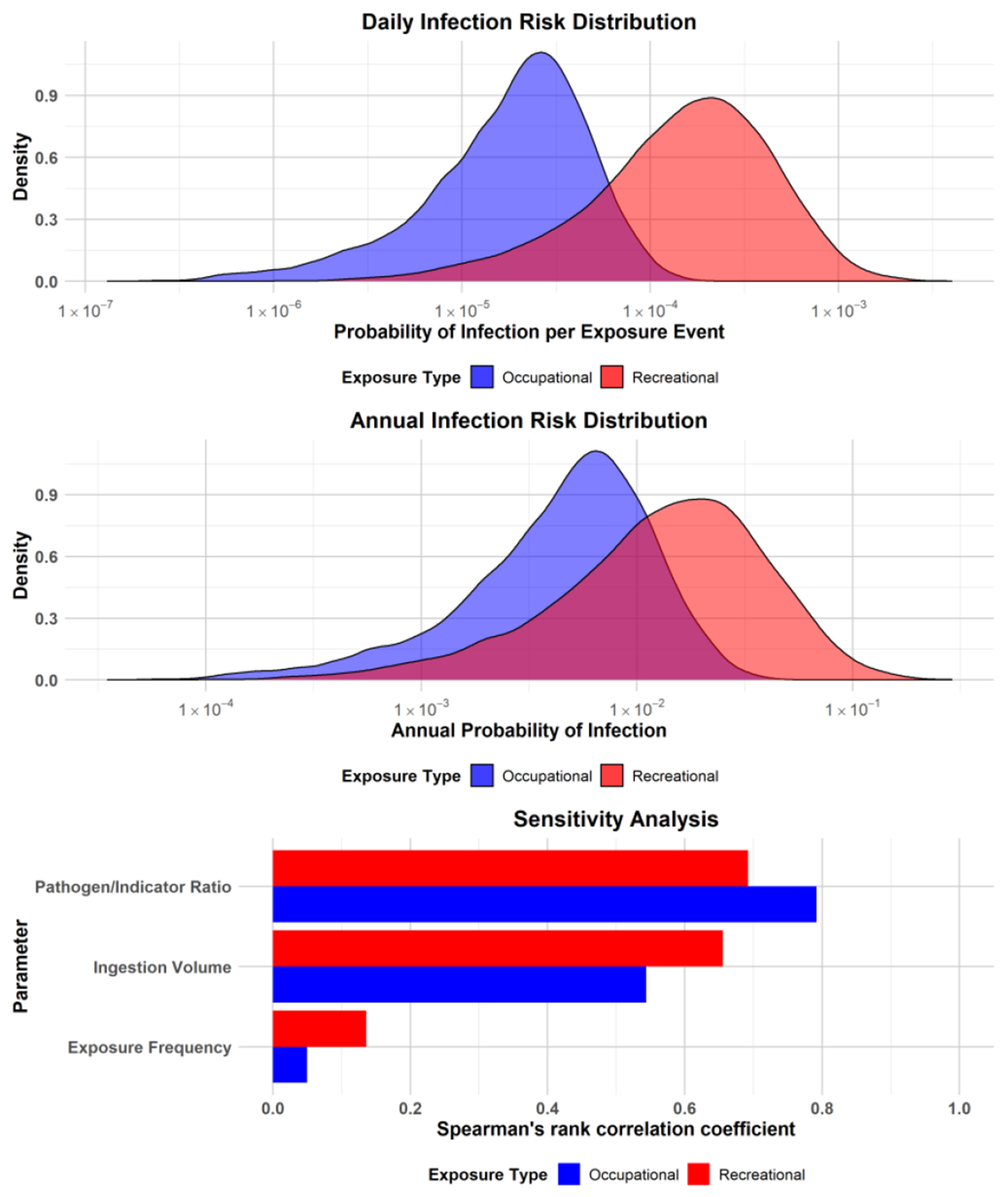
QMRA results for Royal Spring. Top and middle graphs show the daily and annual infection risk distributions. The bottom graph shows the sensitivity analysis using Spearman’s rank correlation coefficients of model parameters for occupational and recreational exposures.

Monte Carlo simulations revealed different risk estimates for occupational and recreational exposures at Royal Spring (Table 2). Recreational users showed a daily infection risk approximately ten times higher than that of occupational exposure, largely due to higher water ingestion volumes during recreational activities. Both estimated scenarios exceeded the WHO reference level of 10^−4^ pppy (Figure 5). Likewise, the calculated median disease burden exceeded the WHO tolerable burden of 10^−6^ DALYs pppy for both exposure scenarios (Table 2). Sensitivity analysis revealed that results were most sensitive to the pathogen-to-indicator ratio, with correlation coefficients of 0.79 and 0.70 for occupational and recreational exposures, respectively (Figure 5). Results were second-most sensitive to the water ingestion volume, showing stronger sensitivity for recreational exposure (0.65) than for occupational exposure (0.54). This difference reflects the higher and more variable ingestion volumes associated with recreational activities.

## 4. Discussion

The results from our year-long monitoring study provide important insights into fecal contamination dynamics and associated health risks in the Royal Spring karst basin. The substantial temporal variability in *E. coli* concentrations (12 to 1732.8 MPN/100 mL) aligns with patterns observed in other karst systems. Mean and median *E. coli* concentrations at Royal Spring for weekly monitoring exceeded the maximum values reported in [71] (14.8–53.0 MPN/100 mL) and [72] (78–99 MPN/100 mL). Additionally, Sinreich et al. [73] found *E. coli* concentrations exceeding 150 CFU/100 mL in karst springs during their monitoring period, with particularly high loads during rainfall events.

The frequent exceedance of water quality standards during the recreation season (37.5% of samples exceeding 240 MPN/100 mL) likely reflects both environmental conditions favorable for bacterial survival and increased sources of contamination during these periods. A study of a karst system in Arkansas (USA) also observed *E. coli* concentrations exceeding recreational water quality standards multiple times, particularly downstream of urban areas [74]. Similar findings were reported in Romania, where five out of six karst springs exceeded regulatory limits for recreational water quality [40]. Our weekly monitoring approach revealed more nuanced temporal patterns than those captured by the monthly or seasonal sampling strategies employed in previous studies, emphasizing the importance of high-frequency monitoring for understanding microbial dynamics in karst systems.

The approximately two-fold higher *E. coli* concentrations during wet-weather conditions at Royal Spring align with patterns documented across various karst systems. Higher microbial loads were found during wet periods in January and April compared to drier conditions in June and November in Romanian karst systems [40], and rapid, pronounced responses to precipitation events at karst springs in Switzerland were typically associated with increases in fecal indicator bacteria [73]. The lack of statistical significance in our wet vs. dry comparison likely reflects our limited number of wet-weather samples. Using a 48-h AMC threshold of 0.5 inches to classify events, most of our sampling occurred during dry periods, making it challenging to statistically validate the observed trend. This sampling limitation is a common challenge in karst systems [75], where capturing storm events requires more intensive sampling programs, particularly during wet-weather periods when contamination risk is typically the highest. More frequent sampling during storm events would likely provide the statistical power needed to confirm this relationship.

The MLR model incorporating discharge, spring temperature, and 48-h AMC explained 23.6% of the variance in *E. coli* concentrations at Royal Spring, with *p* = 0.002. By comparison, Dapkus et al. [44] found that a MLR model using daily average air temperature and 48-h AMC (with hourly precipitation data) from Blue Grass Airport explained 48% of the variance in *E. coli* concentrations at Royal Spring, with *p* < 0.001. However, Dapkus et al. [44] considered *E. coli* concentrations above the upper detection limit or below the lower detection limit as equal to those respective limits, in contrast to our approach of excluding those values. Beyond discharge, temperature, and AMC, several other variables likely influence bacterial dynamics in this system. These include land-use patterns in the watershed [29,30], and sediment transport and resuspension processes, as bacteria can persist while attached to suspended particles [17,18]. Additional factors include variations in groundwater residence times and flow paths through the karst network, which affect bacterial survival and transport [13,14], aging wastewater infrastructure contributing leaks [30], and wildlife activity patterns that influence fecal inputs to the system [28]. The significant relationship between *E. coli* concentrations and spring temperature mirrors findings from the Appalachian karst [75], suggesting enhanced bacterial transport and survival in warmer conditions across karst systems. Similarly, the significant relationship between *E. coli* concentrations and 48-h AMC reflects how karst conduits act as “biologically active conveyors” that can temporarily store and process contaminants between storms [45]. This understanding is crucial for predicting and managing water quality in karst aquifers.

QMRA modeling based on the *E. coli* indicator and pathogen-to-indicator ratios suggested risk patterns that exceeded WHO health benchmarks, with estimated risks being notably higher for recreational exposure than occupational exposure. These findings align with McBride et al. [76], who found that direct exposure to stormwater discharges can pose substantial health risks, particularly during recreational activities. Similar risk levels were reported for recreational exposure in Romanian karst springs (10^−4^ to 10^−3^) [40], suggesting consistent patterns across karst systems. The elevated recreational risks relative to occupational risks in our study can be attributed to higher water ingestion volumes during recreational activities, a key exposure factor also highlighted by [65], indicating swimming-related water ingestion.

The sensitivity analysis identified that modeled risk results were most sensitive to the pathogen-to-indicator ratio. This finding aligns with Korajkic et al. [77], who demonstrated through a comprehensive review that establishing reliable pathogen-to-indicator ratios remains challenging in surface-water systems, largely because of differential survival rates and environmental persistence between indicators and pathogens. Zhang et al. [78] similarly highlighted the importance of accurate pathogen–indicator relationships in QMRA modeling, particularly for systems with multiple fecal inputs. This importance is underscored by studies showing that correlations between pathogens and fecal indicators are commonly statistically significant but weak. For example, while pathogenic *E. coli* detection has been found to correlate positively with indicator *E. coli*, these relationships can be inconsistent across different environmental conditions [79].

The median disease burden calculated from 10,000 Monte Carlo simulations exceeded the WHO’s tolerable burden for both scenarios. Our findings align with Soller et al. [38], who documented elevated risks in recreational waters impacted by multiple fecal source inputs. Fuhrimann et al. [39] similarly found disease burdens exceeding WHO thresholds in their QMRA of surface water exposures, suggesting that elevated pathogen risks may be a common challenge across different hydrologic environments when multiple fecal inputs are present.

## 5. Study Limitations and Implications

While this study provides valuable insights into *E. coli* dynamics and health risks in the Royal Spring basin, several factors warrant consideration when interpreting our results. The absence of molecular detection of the pathogen, combined with the substantial uncertainty demonstrated in our sensitivity analysis, suggests that actual risks may lie between model estimates and non-detection results. The non-detection of pathogenic *E. coli* (*stx1* gene) in our samples could be attributed to an insufficient sample volume to reach detection limits, or the specific targeted pathogens may not have been present. Our weekly sampling frequency may have missed some contamination events, particularly during rapid storm responses characteristic of karst systems. As observed by [67], bacterial concentrations in the Royal Spring basin can change significantly within hours during storm events. Exposure may also have been overestimated: for example, fencing along the first ~180 m of the channel downstream of the Royal Spring orifice limits recreational exposure, although the channel farther downstream is publicly accessible.

Despite these limitations, our findings have important implications for water resource management and public health protection in the study region. The calculated exceedance of WHO health benchmarks for both occupational and recreational exposures indicate the need for improved risk communication and potential access restrictions during high-risk periods. The current treatment practices of Georgetown Municipal Water and Sewer Service include chlorination and filtration. However, wet-period exceedances suggest the need for adaptive measures, such as real-time monitoring to trigger enhanced treatment during high-risk periods. Additionally, our study’s findings highlight the vulnerability of karst aquifers to contamination and the need for comprehensive watershed protection measures. These could include enhanced maintenance of sewage infrastructure, given documented contamination from sanitary sewer leaks [30], implementation of agricultural best management practices, and improved stormwater management in urban areas within the basin. These insights should guide future research efforts and management strategies in the Royal Spring basin and similar karst systems.

## 6. Conclusions

Understanding the dynamics of fecal contamination and associated health risks, especially in mixed land-use settings, can facilitate the development of effective strategies to protect water resources. Our study of the Royal Spring basin, combining long-term monitoring with QMRA, demonstrates the complex interactions between environmental conditions and microbial water quality in karst systems. Our findings emphasize the critical need for adaptive management strategies in karst watersheds and highlight the importance of considering multiple exposure pathways in systems where rapid contaminant transport and variable environmental conditions create complex health risk scenarios. Relatively few studies that integrate water quality assessment and health risk analysis have been conducted for karst systems. Although implementing comprehensive monitoring programs can be resource-intensive, our results highlight the importance of long-term water quality monitoring, especially in the context of changing environmental conditions and multiple water uses. Water managers and researchers should prioritize the development of continuous monitoring systems capable of capturing rapid water quality changes, improve understanding of pathogen–indicator relationships in karst environments, and develop more sophisticated early warning systems for water quality degradation. Such advances would enhance our ability to protect public health while sustainably managing vulnerable karst water resources.

## Supplementary Material

Supplement File

The following supporting information can be downloaded at: https://www.mdpi.com/article/10.3390/w17050745/s1, Table S1: Assay-specific primers, probes and annealing temperatures, and gBlock standard.

## Figures and Tables

**Table 1.** Multiple linear regression results. Bolded *p* values are statistically significant.

Variable	Coefficient	Standard Error	t	*p*
Intercept	−438.63	270.93	−1.619	0.113
Discharge	52.05	93.50	0.557	0.581
AMC 48-h	232.80	102.59	2.269	**0.028**
Temperature	37.65	14.32	2.629	**0.012**
Model Statistics	Value			

R-squared	0.2356			
F-statistic	5.52			
*p*-value	**0.002**			
Residual-standard error	273.6			
Degrees of freedom	41			

**Table 2.** Summary of QMRA results.

	Occupational Exposure			Recreational Exposure		

Risk Metric	Median	95% CI Lower	95% CI Upper	Median	95% CI Lower	95% CI Upper
Daily Infection Risk	2.06 × 10^−5^	1.41 × 10^−6^	8.11 × 10^−5^	1.64 × 10^−4^	1.01 × 10^−5^	9.04 × 10^−4^
Annual Infection Risk	5.11 × 10^−3^	3.49 × 10^−4^	2.02 × 10^−2^	1.45 × 10^−2^	8.93 × 10^−4^	8.03 × 10^−2^
Annual Illness Risk	1.79 × 10^−3^	1.22 × 10^−4^	7.09 × 10^−3^	5.08 × 10^−3^	3.12 × 10^−4^	2.81 × 10^−2^
DALYs	2.33 × 10^−6^	1.59 × 10^−7^	9.21 × 10^−6^	6.60 × 10^−6^	4.06 × 10^−7^	3.65 × 10^−5^

## Data Availability

*E. coli* enumeration data are available from [43]. Precipitation and gauged discharge data are available from [50] and [56], respectively. Other data and results of analyses presented herein are available upon request from the corresponding author.

## References

[1] Global Burden of Disease Collaborators. Global, regional, and national life expectancy, all-cause mortality, and cause-specific mortality for 249 causes of death, 1980–2015: A systematic analysis for the Global Burden of Disease Study 2015. Lancet 2016, 388, 1459–1544. 27733281 10.1016/S0140-6736(16)31012-1 PMC5388903

[2] WHO; UNICEF; World Bank. State of the World’s Drinking Water: An Urgent Call to Action to Accelerate Progress on Ensuring Safe Drinking Water for All; World Health Organization: Geneva, Switzerland, 2022.

[3] Brouwer AF; Masters NB; Eisenberg JNS Quantitative microbial risk assessment and infectious disease transmission modeling of waterborne enteric pathogens. Curr. Environ. Health Rep 2018, 5, 293–304. 29679300 10.1007/s40572-018-0196-x PMC5984175

[4] Devane ML; Moriarty E; Weaver L; Cookson A; Gilpin B Fecal indicator bacteria from environmental sources; strategies for identification to improve water quality monitoring. Water Res. 2020, 185, 116204. 32745743 10.1016/j.watres.2020.116204

[5] Pachepsky YA; Shelton DR Escherichia coli and fecal coliforms in freshwater and estuarine sediments. Crit. Rev. Environ. Sci. Technol 2011, 41, 1067–1110.

[6] Ercumen A; Arnold BF; Naser AM; Unicomb L; Colford JM; Luby SP Potential sources of bias in the use of *Escherichia coli* to measure waterborne diarrhoea risk in low-income settings. Trop. Med. Int. Health 2017, 22, 2–11. 27797430 10.1111/tmi.12978 PMC7169833

[7] Stauber CE; Wedgworth JC; Johnson P; Olson JB; Ayers T; Elliott M; Brown J Associations between self-reported gastrointestinal illness and water system characteristics in community water supplies in rural Alabama: A cross-sectional study. PLoS ONE 2016, 11, e0148102. 26820849 10.1371/journal.pone.0148102 PMC4731071

[8] Harwood VJ; Staley C; Badgley BD; Borges K; Korajkic A Microbial source tracking markers for detection of fecal contamination in environmental waters: Relationships between pathogens and human health outcomes. FEMS Microbiol. Rev 2014, 38, 1–40. 23815638 10.1111/1574-6976.12031

[9] Murphy HM; Prioleau MD; Borchardt MA; Hynds PD Review: Epidemiological evidence of groundwater contribution to global enteric disease, 1948–2015. Hydrogeol. J 2017, 25, 981–1001.

[10] Macler BA; Merkle JC Current knowledge on groundwater microbial pathogens and their control. Hydrogeol. J 2000, 8, 29–40.

[11] Dura G; Pándics T; Kádár M; Krisztalovics K; Kiss Z; Bodnár J; Asztalos A; Papp E Environmental health aspects of drinking water-borne outbreak due to karst flooding: Case study. J. Water Health 2010, 8, 513–520. 20375480 10.2166/wh.2010.099

[12] Ravbar N; Sebela S The effectiveness of protection policies and legislative framework with special regard to karst landscapes: Insights from Slovenia. Environ. Sci. Policy 2015, 51, 106–116.

[13] Pronk M; Goldscheider N; Zopfi J Dynamics and interaction of organic carbon, turbidity and bacteria in a karst aquifer system. Hydrogeol. J 2006, 14, 473–484.

[14] Ford D; Williams P Karst Hydrogeology and Geomorphology; John Wiley & Sons: New York, NY, USA, 2007.

[15] Mahler BJ; Personne JC; Lods GF; Drogue C Transport of free and particulate-associated bacteria in karst. J. Hydrol 2000, 238, 179–193.

[16] Dussart-Baptista L; Massei N; Dupont J-P; Jouenne T Transfer of bacteria-contaminated particles in a karst aquifer: Evolution of contaminated materials from a sinkhole to a spring. J. Hydrol 2003, 284, 285–295.

[17] Gunn J; Tranter J; Perkins J; Hunter C Sanitary bacterial dynamics in a mixed karst aquifer. In Karst Hydrology; Leibundgut C, Gunn J, Dassargues A, Eds.; International Association of Hydrological Sciences Publication 247; IAHS Press: Wallingford, UK, 1997; pp. 61–70.

[18] Marshall D; Brahana JV; Davis RK Resuspension of viable sediment-bound enteric pathogens in shallow karst aquifers. In Gambling with Groundwater—Physical, Chemical and Biological Aspects of Aquifer-Stream Relations; Brahana JV, Eckstein Y, Ongley LK, Schneider R, Moore JE, Eds.; American Institute of Hydrology: St. Paul, MN, USA, 1998; pp. 179–186.

[19] Sherer BM; Miner JR; Moore JA; Buckhouse JC Resuspending organisms from a rangeland stream bottom. Trans. ASAE 1988, 31, 1217–1222.

[20] Sherer BM; Miner JR; Moore JA; Buckhouse JC Indicator bacterial survival in stream sediments. J. Environ. Qual 1992, 21, 591–595.

[21] O’Reilly CE; Bowen AB; Perez NE; Sarisky JP; Shepherd CA; Miller MD; Hubbard BC; Herring M; Buchanan SD; Fitzgerald CC; A waterborne outbreak of gastroenteritis with multiple etiologies among resort island visitors and residents: Ohio, 2004. Clin. Infect. Dis 2007, 44, 506–512. 17243052 10.1086/511043

[22] Kresic N; Stevanovic Z Groundwater Hydrology of Springs: Engineering, Theory, Management, and Sustainability; Butterworth-Heinemann: Oxford, UK, 2010.

[23] Goldscheider N; Chen Z; Auler AS; Bakalowicz M; Broda S; Drew D; Hartmann J; Jiang G; Moosdorf N; Stevanovic Z; Global distribution of carbonate rocks and karst water resources. Hydrogeol. J 2020, 28, 1661–1677.

[24] White WB Geomorphology and Hydrology of Karst Terrains; Oxford University Press: New York, NY, USA, 1988.

[25] Currens JC Kentucky Is Karst Country! What You Should Know About Sinkholes and Springs; Information Circular 4, Series XII; Kentucky Geological Survey, University of Kentucky: Lexington, KY, USA, 2002.

[26] Kentucky Division of Water. Source Water Protection. Available online: https://eec.ky.gov/Environmental-Protection/Water/Protection/Pages/SWP.aspx (accessed on 13 December 2024).

[27] Kentucky State Data Center. Population and Housing Estimates. Available online: http://ksdc.louisville.edu/data-downloads/estimates/ (accessed on 7 December 2020).

[28] Department of Biosystems and Agricultural Engineering, University of Kentucky. Cane Run and Royal Spring Watershed-Based Plan; Report Prepared for U.S. Environmental Protection Agency Under Project Number C9994861-06; University of Kentucky: Lexington, KY, USA, 2011.

[29] Lee SC Identifying Hot-Spots of Fecal Contamination in the Royal Spring Karstshed. Master ’s Thesis, University of Kentucky, Lexington, KY, USA, 2012. Available online: https://uknowledge.uky.edu/ce_etds/2 (accessed on 1 March 2025).

[30] Currens BJ; Hall A; Brion GM; Fryar AE Use of acetaminophen and sucralose as co-analytes to differentiate sources of human excreta in surface waters. Water Res. 2019, 157, 1–7. 30947079 10.1016/j.watres.2019.03.023

[31] Coakley TL; Brion GM; Fryar AE Prevalence of and relationship between two human-associated DNA biomarkers for Bacteroidales in an urban watershed. J. Environ. Qual 2015, 44, 1694–1698. 26436286 10.2134/jeq2014.11.0494

[32] Fryar AE; Currens BJ; Alvarez Villa CS Hydrochemical delineation of spring recharge in an urbanized karst basin, central Kentucky. Environ. Eng. Geosci 2023, 29, 203–216.

[33] Amraotkar AR; Hargis CW; Cambon AC; Rai SN; Keith MCL; Ghafghazi S; Bolli R; DeFilippis AP Comparison of coliform contamination in non-municipal waters consumed by the Mennonite versus the non-Mennonite rural populations. Environ. Health Prev. Med 2015, 20, 338–346. 26068785 10.1007/s12199-015-0472-4 PMC4550604

[34] Baughn C; Bledsoe LA; Groves C Evaluating potential health threats from untreated karst springs as community drinking water sources, Monroe County, Kentucky. In Kentucky Water Resources Annual Symposium Proceedings; Kentucky Water Resources Research Institute, University of Kentucky: Lexington, KY, USA, 2019; pp. 33–34.

[35] Bergeisen GH; Hinds MW; Skaggs JW A waterborne outbreak of hepatitis A in Meade County, Kentucky. Am. J. Public Health 1985, 75, 161–164. 3966622 10.2105/ajph.75.2.161 PMC1645986

[36] Haas CN; Rose JB; Gerba CP Quantitative Microbial Risk Assessment, 2nd ed.; John Wiley & Sons: Hoboken, NJ, USA, 2014.

[37] Howard G; Pedley S; Tibatemwa S Quantitative microbial risk assessment to estimate health risks attributable to water supply: Can the technique be applied in developing countries with limited data? J. Water Health 2006, 4, 49–65. 16604838

[38] Soller JA; Schoen ME; Bartrand T; Ravenscroft JE; Ashbolt NJ Estimated human health risks from exposure to recreational waters impacted by human and non-human sources of faecal contamination. Water Res. 2010, 44, 4674–4691. 20656314 10.1016/j.watres.2010.06.049

[39] Fuhrimann S; Winkler MS; Stalder M; Niwagaba CB; Babu M; Kabatereine NB; Halage AA; Utzinger J; Cissé G; Nauta M Disease burden due to gastrointestinal pathogens in a wastewater system in Kampala, Uganda. Microb. Risk Anal 2016, 4, 16–28.

[40] Stupar Z; Levei EA; Neag E; Baricz A; Szekeres E; Moldovan OT Microbial water quality and health risk assessment in karst springs from Apuseni Mountains, Romania. Front. Environ. Sci 2022, 10, 931893.

[41] Robb K; Null C; Teunis P; Yakubu H; Armah G; Moe CL Assessment of fecal exposure pathways in low-income urban neighborhoods in Accra, Ghana: Rationale, design, methods, and key findings of the SaniPath study. Am. J. Trop. Med. Hyg 2017, 97, 1020–1032. 28722599 10.4269/ajtmh.16-0508 PMC5637580

[42] World Health Organization. Quantitative Microbial Risk Assessment: Application for Water Safety Management; WHO: Geneva, Switzerland, 2016.

[43] Dapkus R Tryptophan-Like Fluorescence and Non-Point Source Pollution in Karst Basins, Inner Bluegrass Region, Kentucky. Master’s Thesis, University of Kentucky, Lexington, KY, USA, 2022. Available online: https://uknowledge.uky.edu/ees_etds/96/ (accessed on 1 March 2025).

[44] Dapkus RT; Fryar AE; Tobin BW; Byrne DM; Sarker SK; Bettel L; Fox JF Utilization of tryptophan-like fluorescence as a proxy for E. coli contamination in a mixed-land-use karst basin. Hydrology 2023, 10, 74.

[45] Husic A; Fox J; Agouridis C; Currens J; Ford W; Taylor C Sediment carbon fate in phreatic karst (Part 1): Conceptual model development. J. Hydrol 2017, 549, 179–193.

[46] Thrailkill J; Sullivan SB; Gouzie DR Flow parameters in a shallow conduit-flow carbonate aquifer, Inner Bluegrass Karst Region, Kentucky, USA. J. Hydrol 1991, 129, 87–108.

[47] Sawyer AH; Zhu J; Currens JC; Atcher C; Binley A Time-lapse electrical resistivity imaging of solute transport in a karst conduit. Hydrol. Process 2015, 29, 4968–4976.

[48] Brugger K World Map of the Köppen-Geiger Climate Classification Updated Map for the United States of America. Available online: http://koeppen-geiger.vu-wien.ac.at/usa.htm (accessed on 13 December 2024).

[49] U.S. Climate Data—Monthly Averages. Available online: https://www.usclimatedata.com/ (accessed on 13 December 2024).

[50] cli-MATE: MRCC Application Tools Environment. Available online: https://mrcc.purdue.edu/CLIMATE/ (accessed on 13 December 2024).

[51] Sims R; Preston D; Richarson A; Newton J; Isgrig D Soil Survey of Fayette County, Kentucky; U.S. Government Printing Office: Washington, DC, USA, 1968.

[52] QIAGEN. DNeasy^®^ PowerWater^®^ Kit Handbook; QIAGEN: Germantown, MD, USA, 2022.

[53] Li B; Liu H; Wang W Multiplex real-time PCR assay for detection of *Escherichia coli* O157:H7 and screening for non-O157 Shiga toxin-producing *E. coli*. BMC Microbiol. 2017, 17, 215. 29121863 10.1186/s12866-017-1123-2 PMC5679507

[54] Herlemann DPR; Labrenz M; Jürgens K; Bertilsson S; Waniek JJ; Andersson AF Transitions in bacterial communities along the 2000 km salinity gradient of the Baltic Sea. ISME J. 2011, 5, 1571–1579. 21472016 10.1038/ismej.2011.41 PMC3176514

[55] Klindworth A; Pruesse E; Schweer T; Peplies J; Quast C; Horn M; Glöckner FO Evaluation of general 16S ribosomal RNA gene PCR primers for classical and next-generation sequencing-based diversity studies. Nucleic Acids Res. 2013, 41, e1. 22933715 10.1093/nar/gks808 PMC3592464

[56] Royal Springs at Georgetown, KY. Available online: https://waterdata.usgs.gov/monitoring-location/03288110/ (accessed on 13 December 2024).

[57] Terry A (Georgetown Municipal Water and Sewer Service, Georgetown, KY, USA). Personal communication, 2024.

[58] Helsel DR Statistics for Censored Environmental Data Using Minitab and R, 2nd ed.; John Wiley & Sons: Hoboken, NJ, USA, 2012.

[59] Kentucky Administrative Regulations. Surface Water Standards. Available online: https://apps.legislature.ky.gov/law/kar/titles/401/010/031/ (accessed on 3 March 2025).

[60] U.S. Environmental Protection Agency. Recreational Water Quality Criteria; Office of Water 820-F-12-058; U.S. EPA: Washington, DC, USA, 2012.

[61] NOAA Online Weather Data (NOWData)—Frequently Asked Questions. Available online: https://www.weather.gov/climateservices/nowdatafaq (accessed on 24 February 2025).

[62] Tooth AF; Fairchild IJ Soil and karst aquifer hydrological controls on the geochemical evolution of speleothem-forming drip waters, Crag Cave, southwest Ireland. J. Hydrol 2003, 273, 51–68.

[63] Kentucky Pollutant Discharge Elimination System Permit No. KYR100000 Available online: https://www.hkywater.org/DocumentCenter/View/183/Kentucky-Pollutant-Discharge-Elimination-System-KPDES-PDF (accessed on 13 December 2024).

[64] R Core Team. R: A Language and Environment for Statistical Computing; Version 4.1.2; R Foundation for Statistical Computing: Vienna, Austria, 2021.

[65] Dufour AP; Behymer TD; Cantú R; Magnuson M; Wymer LJ Ingestion of swimming pool water by recreational swimmers. J. Water Health 2017, 15, 429–437. 28598347 10.2166/wh.2017.255

[66] Pronk M; Goldscheider N; Zopfi J Microbial communities in karst groundwater and their potential use for biomonitoring. Hydrogeol. J 2009, 17, 37–48.

[67] Bandy AM; Cook K; Fryar AE; Zhu J Differential transport of *Escherichia coli* isolates compared to abiotic tracers in a karst aquifer. Groundwater 2020, 58, 70–78. 10.1111/gwat.12889 30982960

[68] Teunis P; Takumi K; Shinagawa K Dose response for infection by *Escherichia coli* O157:H7 from outbreak data. Risk Anal. 2004, 24, 401–407. 15078310 10.1111/j.0272-4332.2004.00441.x

[69] Teunis PFM; Ogden ID; Strachan NJC Hierarchical dose response of *E. coli* O157:H7 from human outbreaks incorporating heterogeneity in exposure. Epidemiol. Infect 2008, 136, 761–770. 17672927 10.1017/S0950268807008771 PMC2870861

[70] Machdar E; van der Steen NP; Raschid-Sally L; Lens PNL Application of Quantitative Microbial Risk Assessment to analyze the public health risk from poor drinking water quality in a low income area in Accra, Ghana. Sci. Total Environ 2013, 449, 134–142. 23416990 10.1016/j.scitotenv.2013.01.048

[71] Frank S; Goeppert N; Goldscheider N Multiple-parameter approach to characterize dynamics of organic carbon, faecal bacteria and particles at alpine karst springs. Sci. Total Environ 2018, 615, 1446–1459. 28935241 10.1016/j.scitotenv.2017.09.095

[72] Vucinic L; O’Connell D; Dubber D; Coxon C; Gill L Multiple fluorescence approaches to identify rapid changes in microbial indicators at karst springs. J. Contam. Hydrol 2023, 254, 104129. 36634484 10.1016/j.jconhyd.2022.104129

[73] Sinreich M; Pronk M; Kozel R Microbiological monitoring and classification of karst springs. Environ. Earth Sci 2014, 71, 563–572.

[74] Covington MD; Gibson KE; Rodriguez J Comparative microbial community dynamics in a karst aquifer system and proximal surface stream in northwest Arkansas. In Arkansas Bulletin of Water Research; Arkansas Water Resources Center, University of Arkansas: Fayetteville, AR, USA, 2018; pp. 3–8. Available online: https://scholarworks.uark.edu/awrcbwr/2 (accessed on 1 March 2025).

[75] Luffman I; Tran L Risk factors for *E. coli* O157 and cryptosporidiosis infection in individuals in the karst valleys of East Tennessee, USA. Geosciences 2014, 4, 202–218.

[76] McBride GB; Stott R; Miller W; Bambic D; Wuertz S Discharge-based QMRA for estimation of public health risks from exposure to stormwater-borne pathogens in recreational waters in the United States. Water Res. 2013, 47, 5282–5297. 23863377 10.1016/j.watres.2013.06.001

[77] Korajkic A; McMinn BR; Harwood VJ Relationships between microbial indicators and pathogens in recreational water settings. Int. J. Environ. Res. Public Health 2018, 15, 2842. 30551597 10.3390/ijerph15122842 PMC6313479

[78] Zhang Q; Gallard J; Wu B; Harwood VJ; Sadowsky MJ; Hamilton KA; Ahmed W Synergy between quantitative microbial source tracking (qMST) and quantitative microbial risk assessment (QMRA): A review and prospectus. Environ. Int 2019, 130, 104703. 31295713 10.1016/j.envint.2019.03.051

[79] Ferguson AS; Layton AC; Mailloux BJ; Culligan PJ; Williams DE; Smartt AE; Sayler GS; Feighery J; McKay L; Knappett PSK; Comparison of fecal indicators with pathogenic bacteria and rotavirus in groundwater. Sci. Total Environ 2012, 431, 314–322. 22705866 10.1016/j.scitotenv.2012.05.060 PMC3587152

