# Supplementary material for: Quantifying Temporal Dynamics of E. coli Concentration and Quantitative Microbial Risk Assessment of Pathogen in a Karst Basin
Source: Water (Basel). Author manuscript; Available in PMC 2025 Aug 22. (PMC12369636; doi:10.3390/w17050745)
Supplement: Supplement File [file NIHMS2077959-supplement-Supplement_File.pdf]

Table S1: Assay-specific primers, probes and annealing temperatures, and gBlock standard.

Primer Name	Target	Region	Direction	Sequence 5'-3'	Target Length	Annealing Temp (°C)	Standard
341F	16S rRNA	V3-V4	Forward	CCT ACG GGN GGC WGC AG	444	55	gBlock
805R			Reverse	GAC TAC HVG GGT ATC TAA TCC			
STEC_stx1-F	*E. coli* O157:H7 (STEC)	*stx1*	Forward	GGA TTT CGT ACA ACA CTG GAT GAT	67	60	gBlock
STEC_stx1-R			Reverse	ATC CAC ATC TTC AGC AGT CAT TAC A			
STEC_stx1-P (FAM)			Probe	/56-FAM/CAG TGG GCG TTC TT/3MGBEc/			

**Target**	**gBlock Sequence 5'-3"**
STEC_stx 1 gBlock	TCCTACACGAACAGAGTCTTGTCCATGATAATCAGGCAGGACACTACTCAACCTTCCCCAGTTCAATGTAAGATCCA CATCTTCAGCAGTCATTACATAAGAACGCCCACTGAGATCATCCAGTGTTGTACGAAATCC
